# Activating the knowledge-to-action cycle for geriatric care in India

**DOI:** 10.1186/1478-4505-9-42

**Published:** 2011-12-02

**Authors:** Jenna M Evans, Pretesh R Kiran, Onil K Bhattacharyya

**Affiliations:** 1 Institute of Health Policy, Management and Evaluation, University of Toronto, 155 College Street, Suite 425, Toronto, ON M5T 3M6, Canada; 2 Department of Community Health, St. John's Medical College, Sarjapur Road, Bangalore 560034, India; 3 Li Ka Shing Knowledge Institute, 30 Bond Street, First Floor, Toronto, ON M5B 1W8, Canada

## Abstract

Despite a rapidly aging population, geriatrics - the branch of medicine that focuses on healthcare of the elderly - is relatively new in India, with many practicing physicians having little knowledge of the clinical and functional implications of aging. Negative attitudes and limited awareness, knowledge or acceptance of geriatrics as a legitimate discipline contribute to inaccessible and poor quality care for India's old. The aim of this paper is to argue that knowledge translation is a potentially effective tool for engaging Indian healthcare providers in the delivery of high quality geriatric care. The paper describes India's context, including demographics, challenges and current policies, summarizes evidence on provider behaviour change, and integrates the two in order to propose an action plan for promoting improvements in geriatric care.

## Introduction: an aging India

The size of India's older adult population is greater than the total population of many developed and developing countries. According to *World Health Statistics 2011*, 83 million persons in India are 60 years of age and older, representing over 7% of the nation's total population [1]. Over the next four decades, India's demographic structure is expected to shift dramatically from a young to an aging population resulting in 316 million elderly persons by 2050 [2]. The aging population is a sign of successful development in medical sciences and technology, living standards, and education, but the elderly also raise unique social, economic, and clinical challenges, including a growing demand for increasingly complex healthcare services. Chronic diseases now constitute the leading cause of death and disability among India's old in both urban and rural areas [3,4].

Despite an aging population, geriatrics - the branch of medicine that focuses on healthcare of the elderly - is relatively new in India with many practicing physicians having little knowledge of the clinical and functional implications of aging [5,6]. According to the World Health Organization's (WHO) multi-country study, *Integrated Response of Health Systems to Rapidly Ageing Populations*, India's old, their caregivers, and healthcare providers passively accept ill-health as part of old age [7]. In fact, healthcare providers often view elderly patients in a "negative and mechanistic fashion" [6]. Condemnatory attitudes and limited awareness, knowledge or acceptance of geriatrics as a legitimate discipline can manifest in inaccessible or poor quality care. For example, elderly persons in India often die from preventable conditions like bronchitis, asthma, and pneumonia [8]. India's old are hospitalized for an average of 32 days, often due to inadequate community-based health and social support rather than ongoing acute needs [9]. This population takes an average of six prescription drugs concurrently [10,11] and often suffer from adverse drug reactions [12,13]. An absence of human and institutional capacity for geriatric care in the Indian healthcare system contributes to variations in morbidity and access to care based on gender, location, and socioeconomic status [14].

Attitudes and practices that fail the elderly may be reinforced by cultural values that reject long-term hospitalization of the old because it is viewed as a sign of disrespect; traditionally younger family members tend to the needs of their elderly relatives. In a study of bedridden elderly patients in northern India, 82% of the primary caregivers were relatives and untrained hired help was frequently sought [15]. Preventable medical complications like urinary tract infection and pressure ulcers occurred in over 39% of patients, and caregivers reported burn-out and need for respite [15]. Changes to India's economy are influencing the availability of informal care for the elderly. Urbanization and industrialization have resulted in increased migration of the young to urban areas, improved female employment opportunities, and a shift in values towards commercialism and individualism, all of which have contributed to lower fertility rates, a breakdown in social support networks, disintegration of families, and in turn fewer informal caregivers [16,17]. At least 5% of elderly persons in both urban and rural areas live alone [18]; some reports suggest that as many as 30% either have no family to live with or are unable to reside with family members for various reasons [19].

Dramatic demographic, social, and economic shifts in India have created an urgent need for good quality medical and social care for the nation's elders. However, meeting the needs of an aging population in a resource-constrained environment characterized by limited awareness, knowledge, and research in geriatrics is challenging. Improved research production and knowledge uptake - processes referred to collectively as knowledge translation - can help determine how best to intervene. Knowledge translation is defined as a dynamic and iterative process that involves the synthesis, dissemination, exchange, and application of knowledge to improve health status, provide more effective services, and strengthen the healthcare system [20]. The aim of this paper is to argue that knowledge translation is a potentially effective tool for engaging healthcare providers in the delivery of accessible, high quality geriatric care in India. The paper is divided into three sections: (1) "Geriatric care in India," which describes the context, including demographics, challenges, and current policies and perspectives; (2) "Knowledge translation: a way forward?," which summarizes research on changing the behaviour of healthcare providers from both general and geriatric-specific viewpoints; and (3) "Towards a knowledge translation action plan for India," which integrates what we know about geriatric care in India with what the literature on knowledge translation recommends. The purpose of this paper is not to advocate for a specific intervention, but rather to argue for the potential benefits of utilizing knowledge translation theories and strategies to address the problem of poor quality geriatric care in India.

## Geriatric care in India

India is a lower-middle income country with a gross domestic product (GDP) per capita of $2,930 at purchasing power parity [21]. In recent years, health expenditure as a percentage of GDP has hovered around 4.2% [21]. Private health spending accounts for more than 70% of all health spending, the majority of which is out-of-pocket at the point of service [22]. Even though community-based health insurance schemes show promise, less than 20% of Indians have some form of health insurance; nevertheless, a large portion of the population choose to bypass free public services to pay out-of-pocket in private institutions [23-25]. Accessibility plays a role in the preference for private care: most facilities and providers operate in the private sector and public health infrastructure is not evenly distributed across India's states. Both sectors face critical challenges. A lack of staff, drugs, and equipment plague the public system, while the private sector is largely unregulated with serious complaints regarding poor quality of care and unethical behaviour [24]. At least 36 million people in India fall below the poverty line each year as a result of healthcare costs [26]. Due to their financial dependence, elderly persons are among the most vulnerable. Within the context of these issues, the country carries a "double disease burden" of both non-communicable and infectious illnesses [14]. However, government health expenditure is slated to increase by 1% of GDP over the next 5 years, which will provide additional resources to address both sets of healthcare needs [27].

Although this paper adopts a broad perspective in examining the status of geriatric care in India, the nation consists of twenty-eight states and seven union territories with considerable heterogeneity in demographics, disease burden, and healthcare coverage [2,28]. A state-specific discussion is beyond the scope of this paper, but our analysis and recommendations incorporate consideration for local and regional differences.

### Many aging faces

India's elders, aged 60 and over, make important contributions to society not only via the formal workforce (primarily in agriculture), but also in raising grandchildren, volunteering, caring for the sick, resolving conflict and offering counsel, and translating experience, culture, and religious heritage [7]. However, delivering quality healthcare services to this population has proved challenging for a number of reasons. The elderly in India are a heterogeneous population with variations in morbidity across several dimensions, gender, location and socioeconomic status in particular, as well as great diversity in cultures, religions, and languages. At least 65% of India's old live in rural areas and are illiterate and economically dependent [5,18]. Cardiovascular diseases, respiratory disorders, hearing and visual impairments, depression, and infections such as tuberculosis are common [8]. Furthermore, this population is characterized by irregular utilization of healthcare services due to inaccessibility, immobility, misconceptions, and poverty [14]. While some choose to self-medicate or use home remedies, the majority report that they do not seek treatment because their ailment is "not serious" (32-50%) or because of financial constraints (20-28%) [18]. Healthcare utilization is greater among older adults with higher levels of education, among those living in urban rather than rural areas, and among those seeking treatment for communicable rather than non-communicable diseases [18,28]. Non-compliance with treatment plans and drug regimens is also an impediment to managing the health of this population, resulting in an estimated 8% of hospital admissions; the most commonly cited reasons for non-compliance include cost, inadequate instruction, and switch to non-conventional treatment [13]. The influence of cost constraints on decisions to seek or continue treatment, as noted above, highlights the fundamental role poverty plays in shaping the health of India's aging population. Any attempt to improve the quality of geriatric care and outcomes in India must address or account for these financial barriers to access. In fact, planners and policymakers should take note of signs of gradual change, such as increasing literacy levels and intergenerational distance in interactions; in the future the elderly will demand more financial, social and healthcare services than the present generation [29].

### Geriatric health services and providers

Although aging research in India is relatively new, the Geriatric Society of India, the Indian Academy of Geriatrics, and the Association of Gerontology are established institutions dedicated to the cause [30]. Unfortunately, most members lack formal training in geriatrics [21]. Non-governmental organizations (NGOs) include HelpAge India, the Agewell Foundation, and the Dignity Foundation, among others [7]. Government and social policies for the elderly in India are also growing and include old age pensions, the National Policy for Older Persons (1999), the National Initiative on Care for the Elderly (2004), and the Maintenance and Welfare of Parents and Senior Citizens Act (2007). Despite the importance of these initiatives in directing attention towards the needs of the elderly, criticisms and challenges to implementation abound. Efforts across NGOs, government, and provider organizations tend to be uncoordinated, rely heavily on NGOs even though they are not evenly distributed across India, and have been described as "wish lists" that do not incorporate financial considerations or mechanisms for monitoring performance [31-33]. Others highlight the lack of attention to gender differences and biases towards the needs of those residing in urban areas and retiring from the formal work sector [33].

The most recent national policy effort is the National Programme for the Health Care of the Elderly (NPHCE), released in early 2011. Until now, healthcare delivery options for geriatric care and associated knowledge and skill requirements have not been accorded due attention. Health service organizations and individual providers often fail to prioritize elderly patients, do not provide continuity of care, and have limited human and material resources to manage chronic conditions [7]. Few facilities are dedicated exclusively to geriatric care; those that are tend to be concentrated in urban areas and are prohibitively expensive [6]. General hospitals lack the capacity to deliver comprehensive geriatric care to the elderly [6] and although some hospitals provide geriatric outpatient services, there are very few geriatric inpatient units even though the Indian Council of Medical Research (ICMR) states that the special needs of the elderly are best dealt with by a geriatric unit with trained geriatricians and nursing staff [34]. Furthermore, old-age homes, day-care centres, and mobile medicare units number in the hundreds. These facilities are managed by NGOs or funded partially by government, but tend to be urban based, expensive, or focused on tertiary as opposed to primary care, leaving many of India's 83 million seniors without appropriate healthcare [14,35]. Several indigenous systems of medicine also operate amidst the formal public and private systems, and offer treatments which may be more accessible, affordable or acceptable to the rural elderly [36,37]. However, a recent study suggests that graduates of indigenous medical programs often lack the clinical training required to utilize diagnostic tools, conduct basic procedures, and handle primary care emergencies [38].

Indian researchers and experts, including the ICMR, have reached consensus on the need to educate and train healthcare providers in geriatrics, and to develop gender-sensitive and rural-based geriatric services that operate through the existing primary healthcare system [5,7,14,29,39,40]. With a network of at least 2,000 community health centres and 22,000 primary health centres in rural India, the infrastructure needed to deliver geriatric care exists, but the required human resource capacity does not. A WHO India project series on community-based healthcare for the elderly confirms this point [41]. The total number of physicians and nurses in India is less than half the WHO benchmark of 25 workers per 10,000 population [42]. Many community-based facilities lack medical and paramedical personnel; for example 18% of primary health centres function without a doctor and 16% without a pharmacist [22]. Healthcare workers are unevenly distributed with more workers practicing in southern states and in urban areas than in northern states and rural areas [42]. Many healthcare workers - both informal and formal - have no medical training [42]. In order to improve geriatric care, India needs a national human resource policy to address issues of provider shortages, distribution, education, and quality [42] as well as training opportunities to sensitize healthcare workers to the aging process [7,40]. Community volunteers and medical, paramedical, and indigenous providers must be trained to identify issues common among elderly patients, to conduct comprehensive surveys of morbidity and functional status, and to engage in capacity-building of geriatric services in local communities [5,41]. In hospitals, collaborative training among physicians, psychiatrists, nurses, dentists, urologists, and other professionals can foster multi-disciplinary approaches to geriatric care [5]. Unfortunately, there are few opportunities for geriatric knowledge-building and formal training in India. Only one of the country's 206 medical colleges, Madras Medical College, has a full-time geriatric MD program. Indira Gandhi National Open University offers a one-year part-time Post-Graduate Diploma in Geriatric Medicine with 4-weeks of practical training to doctors working in different streams of medicine. Increasing the availability of specialized education and training opportunities in geriatrics is important considering the inadequacy of general healthcare programs in raising awareness of issues related to healthy aging. In a recent study of senior-level students from medical, nursing, and social work colleges in India, about 50% were unaware of policies relating to the health and well-being of the elderly and none of the students demonstrated recognition of the clinical and functional implications of aging [43].

The NPHCE seeks to address many of the identified gaps in institutional and human capacity for the provision of good quality geriatric care. Compared to previous policy-driven attempts at change - most notably the National Policy on Older Persons - the NPHCE is more action and results-oriented. The program aims to "provide accessible, affordable, and high-quality long term, comprehensive and dedicated services to an aging population" by (1) expanding infrastructure to include Regional Geriatric Centres, geriatric units in district hospitals, and community-based geriatric clinics; (2) establishing specialized geriatric training programs and research institutes; and (3) utilizing mass media to educate the public [44]. The NPHCE outlines the source, purpose, and flow of funds; the responsibilities and inter-dependencies of various players at the national, regional, and local levels; and requirements for staff mix as well as data collection and reporting. In accordance with expert opinion, the program also promotes strong inter-organizational linkages and referral mechanisms as well as training and support for informal caregivers [7]. The level of detail promotes a standardized approach to implementation which will help improve the consistency of service availability and quality. However, the NPHCE may also be viewed negatively as a top-down initiative that leaves little room for adaptation based on local needs and preferences [45]. That being said, states will have the flexibility to shift up to 10% of allocated funds, and will take over responsibility from the central government once units are fully functional. The program is being implemented in phases beginning with 100 districts in 21 of India's 28 states.

### Summary

The literature on geriatric care in India summarizes demographic trends, the medical and socioeconomic problems among the elderly, general provider characteristics, contextual considerations, and suggestions for improvement with a particular emphasis on education and training as well as building on India's existing primary healthcare system. In general, the literature reviewed adequately answers the "what" and the "why" of aging and poor geriatric care in India, but more research is needed to understand "how" to instigate improvements. For example, while many papers contain descriptive or normative discussions from knowledgeable authors, little empirical research exists to support their recommendations.

## Knowledge translation: a way forward?

Knowledge Translation (KT) is the scientific study of the methods for closing the knowledge-to-practice gap and the analysis of barriers and facilitators inherent in this process. KT is based on the premise that quality of care and patient outcomes improve when research findings are translated into practice. However, KT is not only about translating and utilizing evidence, but also about the process of producing knowledge. Graham et al.'s [20] conceptual framework for KT synthesizes several international frameworks and was adopted by the Canadian Institutes for Health Research. The defining feature of the framework is that it captures both the knowledge creation and action components of the "knowledge-to-action" cycle. Knowledge creation consists of research inquiry, the synthesis of evidence, and the production of knowledge-based tools and products, such as clinical practice guidelines. The action cycle consists of problem identification, adaptation of the knowledge to the context, implementation of the intervention, evaluation, and sustaining changes. KT differs from continuing medical education (CME) in that CME focuses on enhancing clinical competence and maintaining certification, whereas KT involves broader activities and goals including behaviour change and improved health outcomes [46]. KT can also involve other stakeholders including policymakers, researchers, community members, managers, and patients, in addition to clinicians.

While Graham et al.'s [20] framework provides an overview of KT, a model designed by Farkas et al. [47] provides an examination of specific KT strategies aimed at improving elder care. Interventions operate at four levels: the individual health professional, healthcare teams, organizations providing healthcare, and healthcare systems. Farkas and her colleagues are interested in instigating change at the individual level. As such they identify three target populations: researchers, providers and administrators, and consumer and families. They outline four strategies and their corresponding goals: (1) exposure (to increase knowledge), (2) experience (to increase knowledge and positive attitudes), (3) expertise (to increase competence) and (4) embedding (to increase utilization over time). Under each strategy are a variety of KT interventions that can be used to achieve the corresponding goals depending on the audience of interest. Exposure strategies are the most passive methods of dissemination and for providers include activities like reading publications, participating in conferences, and accessing web-based resources. The strategies become increasingly complex from experience to embedding and involve provider-centered KT interventions such as mentorship, practice visits, training programs, supervision, technical assistance, and feedback tools. The micro-level perspective of the Farkas et al. framework, which helps us identify problems, clarify goals, and select appropriate interventions complements the broader knowledge-to-action framework presented by Graham and colleagues, which outlines the steps we need to take to introduce and sustain change [20,47].

### State of the evidence

Most developed countries are farther along in the demographic transition than developing countries; this creates a practical learning opportunity for countries slated to become "aging nations" like India and China. Even in the US few opportunities exist for medical students and physicians to receive geriatric training; as a result "many professionals are poorly skilled in caring for older adults' unique needs" [48]. Physicians in the US experience difficulty in caring for the elderly because of administrative burden, medical complexity, and interpersonal challenges; researchers suggest that changes in the care delivery system and medical education are required [49]. KT can help facilitate evidence-based change across various systems grappling with the problem of poor geriatric care.

There is a wide range of KT interventions targeting providers, organizations and health systems, such as interactive educational sessions, audit and feedback, reminders, and pay for performance. In addition, interventions can be patient or family mediated, technology enabled, or multi-faceted, combining 2 or more modalities. Reviews of KT interventions consistently demonstrate moderate improvements in care in high, middle, and low-income settings [50-54]. For example, in a systematic review of 102 interventions, practice visits, patient-mediated interventions, and multi-faceted interventions were effective; audit and feedback and reminders produced mixed results; and educational materials, conferences, and workshops had no impact [53]. Similarly, in low-resource settings the simple dissemination of written guidelines is often ineffective, but audit and feedback as well as multifaceted interventions are generally effective [55]. Although reviews from developing countries sometimes reach different conclusions from reviews of studies in wealthier settings [55], performance feedback and educating patients were also successful in changing the knowledge, attitudes, and/or behaviours of providers in the US [56]. In general, these studies suggest that single methods with no additional follow-up fail to effect change.

Unfortunately very few KT papers address geriatric care; those that do hail from the developed world and tend to focus exclusively on CME [47,56,57]. A recent scoping review found a scarcity of KT literature pertaining to geriatric care; out of 53 systematic reviews of KT research, only two focused on KT in the care of older adults [58]. These two reviews found that KT can influence physician behaviour and patient outcomes particularly when multifaceted interventions are utilized.

KT research and practice is often narrow in scope, targeting a specific clinical setting or group of providers. However, KT can also be an effective tool for promoting changes on a larger scale. In fact there is increasing support for KT at international and national levels. Two examples include the WHO's Evidence-Informed Policy Networks (EVIPNET) currently active in Asia, Africa, and the Americas, and the Regional East African Community Health (REACH) Policy Initiative [59,60]. Collaborative KT interventions that involve multiple stakeholders, such as these, enhance the probability of large-scale evidence-informed changes in low-resource settings [61,62].

### KT for geriatric care in India

There are five key reasons why KT may be an effective tool for improving the quality of geriatric care in India. First, as demonstrated above, evidence suggests that KT *can *improve quality of care, in part, by changing the behaviors of providers. Despite a lack of geriatric KT research within India, unpublished data reveal an increase in physician confidence in elder care after a one-year geriatric training program at the Indira Gandhi National Open University [31]. Second, local evidence suggests gaps in the production, translation, and uptake of knowledge. For example, several authors advocate for the development of geriatric education, training, and research programs in India [5,30,39,63]. It has also been reported that research findings with implications for the welfare of the elderly never reach Indian healthcare providers, caregivers, or policymakers [30]. Therefore, one of the many barriers to improvements in geriatric care in India appears to be a lack of adequate KT. Third, a KT approach aligns with expert consensus in India by including geriatric education and training efforts. However, KT also goes beyond these traditional methods of instigating change from within the classroom. This is important because poor performance is not always the result of a lack of knowledge or skills [64]. For example, providers often feel pressure from patients and communities to provide inappropriate treatments. KT acknowledges these contextual factors and promotes the targeting of multiple stakeholders. Furthermore, some scholars advocate for an elder care model that emphasizes a family and community care system [16,65]. A KT perspective is in line with this proposition. Successful KT requires attention to all relevant stakeholders; in India, both formal and informal caregivers are of interest. In a resource-constrained, family-oriented environment, the role of informal caregivers cannot be overlooked. Fourth, a geriatric KT strategy can strengthen and extend the recently initiated NPHCE. Previous national efforts to improve elder care have been criticized for not being implemented universally or effectively. Considering that much of the NPHCE focuses on capacity-building through education, training, and research, KT frameworks and methods are relevant and may increase the probability of successful implementation and sustainable improvements. Finally, in the near future the case for shifting more resources to geriatric care and chronic disease will intensify. According to the Disease Control Priorities Project in India, it is estimated that 1 million lives could be saved using interventions targeted at non-communicable illnesses like cardiovascular disease and cancer [27]. The costs associated with addressing these growing needs are within the projected increases in health spending planned by the Indian government [27]. As the number of elderly persons continues to increase and as India experiences unprecedented economic growth the demand for high quality geriatric care, including chronic disease management, will escalate.

## Towards a knowledge translation action plan for India

**Figure 1:**
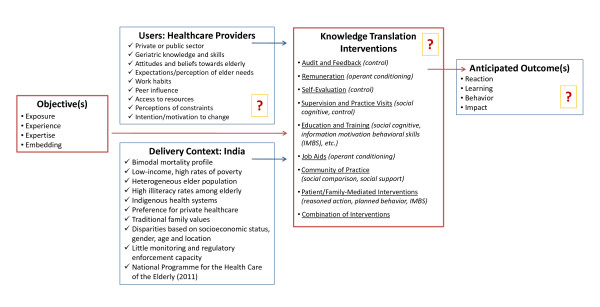
**Towards an Action Plan for Geriatric Knowledge Translation in India**.

Five key questions must be addressed in order to initiate action in KT [12]: (1) What should be disseminated?, (2) To whom?, (3) By whom?, (4) How should it be disseminated?, and (5) What is the potential impact? Figure 1 illustrates the answers to many of these questions and depicts areas where more research is needed (symbolized by question marks) as well as the pathway from objectives to anticipated outcomes.

### What should be disseminated?

Research evidence from the fields of geriatrics and gerontology need to be disseminated to India's healthcare providers. Geriatrics is the study of health and disease in later life and emphasizes comprehensive care for older persons as well as the well-being of their caregivers. Gerontology is the study of the aging process and involves the study of the physical, mental, and social changes that occur as people age. The production, dissemination and sharing of knowledge from both fields are required in order for healthcare providers to deliver high quality services to India's elderly. In the context of a poverty-stricken population, best practices must be identified and applied with consideration for financial barriers that may prevent some elderly patients from seeking and complying with treatment, lifestyle, and medication regimens.

In planning dissemination efforts, it is important to begin by clarifying goals. As described above and depicted in Figure 1, exposure, experience, expertise, and embedding constitute four distinct and increasingly complex KT objectives [47]. Considering reports that suggest low knowledge and awareness of the clinical and functional implications of aging among India's providers, it is appropriate to initiate KT work in the areas of exposure and experience, but to build a plan for incorporating more complex strategies associated with expertise and embedding, such as providing performance feedback and developing communities of practice. Furthermore, the variability in cost and effect of KT interventions and our lack of data on overall cost-effectiveness make an incremental approach with an initial focus on exposure and experience strategies a prudent first step.

### To whom should it be disseminated?

Although the target recipients of KT efforts can span multiple stakeholder groups, this paper focuses primarily on formal healthcare providers, which includes doctors, nurses, community workers like home health assistants and paramedicals, and even those with formal training in traditional systems of medicine such as Ayuverda, Yoga, Unani, Sidda, and Homeopathy (AYUSH). Figure 1 outlines some of the user group characteristics that we need to better understand as well as contextual factors. Models of provider behaviour can be used as a supplement; Brugha and Zwi [64], for example, outline considerations regarding national context; needs, expectations, and social environment; provider knowledge and attitudes; and patient-provider interactions as well as potential KT interventions at the community and policy levels.

### By whom should it be disseminated?

India's geriatric KT initiatives can be planned, organized, and implemented jointly by national and state governments using the structures and relationships established through the NPHCE. For example, the NPHCE calls for the appointment of liaisons to manage relationships among various layers of government and service organizations. These individuals could take on responsibilities similar to those of a knowledge broker by facilitating information flow and awareness of relevant research evidence across multiple stakeholder groups [62]. Other entities involved in elder care such as the Geriatric Society of India, the Indian Academy of Geriatrics, the Association of Gerontology, the ICMR, and NGOs such as HelpAge India should also be included in KT efforts; their expertise, networks, and resources can be used to support KT across organizational, professional, and political boundaries.

Considering the heterogeneity in demographics, disease burden, and healthcare coverage across states, there will be no "one size fits all" intervention for geriatric care improvement. Therefore collaborative approaches that promote a balance between standardization of KT methods and content, and flexibility for local adaptation are essential. A national human resource policy to improve the availability, distribution, education, and quality of healthcare providers is fundamental to the success and sustainability of these efforts.

### How should it be disseminated?

Many methods exist for transferring knowledge; the most common are listed in Figure 1 along with their associated theories [66]. It is important to link methods with theory so that interventions can be designed around an existing body of knowledge. We lack information regarding which factors influence the effectiveness of different interventions; the use of theory in the development and implementation of KT interventions will allow for more accurate interpretations of why certain methods have positive or negative effects [67,68]. Based on the available evidence, education and training methods must be used in combination with KT strategies that promote active-mode learning and application [50-53,55-57]. In particular, social interaction methods, such as communities of practice and frequent contact with a supervisor, mentor, knowledge broker, or opinion leader show promise. However, most behavioural interventions have been developed for use with individuals motivated to seek help [67]; thus, for healthcare providers lacking the desire to change, KT strategies that target the patient and caregiver or broader organizational or system environment may also be required. Both options are already underway in India. The NPHCE details several organizational and system-level changes to healthcare delivery, and incorporates education efforts via mass media and home visits to build capacity for informal and self care. These strategies address the problem of limited knowledge and awareness from multiple perspectives, and can help support provider behaviour change.

The targeted healthcare providers will have varied levels of knowledge and skill in geriatrics and gerontology, and potentially divergent views of elder care, depending on their role, setting, training, and past experiences [67]. KT is a user-focused endeavour in which both the content and the method must be tailored to the needs, capabilities, and contexts of different stakeholder groups. In order to undertake this process effectively, the geriatric-related knowledge, skills, attitudes, beliefs, and perceptions of various healthcare providers in India need to be better understood, as depicted in Figure 1. In addition to being developed with consideration for provider characteristics and the factors that influence provider behaviour, KT interventions to improve geriatric care in India must also be context-specific and inexpensive in order to offer sustainable benefits to patients and their families.

### With what effect should it be disseminated?

Expected outcomes of KT interventions are divided into four levels of evaluation [69]: (1) Reactions are measures of participant views of the intervention, including satisfaction, level of participation, attitude, and confidence; (2) Learnings are measures of what the participants have learned from the intervention, including intention to apply learning or change practice; (3) Behaviours are measures of whether new knowledge is being applied in practice; and (4) Impacts are measures of change in individual or organizational performance measures, and/or change in patient outcomes. Although measurement and reporting requirements are included in the NPHCE, the focus is on monitoring implementation in terms of physical progress and financial targets. Systematically collecting information using a set of indicators from each of the four levels of evaluation outlined above will offer additional insights into whether the program is having the desired impact on human capacity and patient outcomes. Successful infrastructure development and program implementation are not ends in themselves; they are means to an end. Performance measurement must therefore reflect other outcomes such as provider behaviour change and the ultimate goal of improved quality of care for the elderly.

### Knowledge Production

Although KT research often focuses on the action cycle, the knowledge creation cycle precedes action and requires particular attention in India. Proven techniques and approaches can be borrowed from around the world, but their value in India is questionable. There is a need for more research on cost-effective approaches to elder care from within the Indian context [5,16]. India has already reduced the cost of heart and cataract surgeries to 10% of the cost in the US [70]; similar frugal innovations may make geriatric care more broadly available, affordable, and appropriate.

The NPHCE proposes the establishment of Regional Geriatric Centres (RGCs) at each of the eight existing Regional Medical Institutes in India. In addition to providing tertiary services, the RGCs will offer continuing education and training programs, and will conduct research in geriatrics and gerontology to develop evidence-based service and treatment protocols. Funds for these activities are available from a grant under the NPHCE as well as from national and international agencies.

Areas of elder research that have been well-covered in India include caregiving, social supports, demographic changes, widowhood, and intergenerational interactions whereas mental health, elder abuse, health behaviours, and human resource issues require further attention [30]. The three basic functions of aging research are: (1) to provide basic data on the overall status and needs of the elderly, (2) to understand what constitutes good quality of life for the elderly, and (3) to formulate, execute, and evaluate appropriate interventions to improve elder care [30]. Evaluation is of particular importance because KT intervention evaluations in low-and-middle income countries tend to be less rigorous [64] and because the ability to pay for KT efforts is lower in these contexts, their value must be demonstrated. Many initiatives in India have potential for widespread use, or at the very least can contribute to learning, but they need to be systematically documented, monitored, and assessed. A systematic approach to knowledge production and translation requires a common language and understanding of the purpose and process of KT among designers, adopters, and reviewers. A common framework and language is important because they help researchers replicate studies and determine how intervention content influences effectiveness. Ideally researchers should report on all of the following aspects of any given intervention: content or elements of the intervention, characteristics of those delivering the intervention, characteristics of the recipients, the setting, the mode of delivery, the intensity and duration of the intervention, and adherence to the delivery protocols [66].

## Conclusions

As a result of dramatic demographic, social, and economic shifts, India's growing elderly population needs high quality medical and social care. However, current literature suggest that negative attitudes, and limited awareness, knowledge, and acceptance of geriatrics as a legitimate discipline result in inaccessible and poor quality care for India's old. Competing demands, the large numbers requiring support, and resource availability remain important challenges. Improved research production and knowledge uptake in geriatrics and gerontology are required. KT is a promising tool for engaging healthcare providers in the delivery of high quality geriatric care.

India's NPCHE sets the foundation for a comprehensive, long-term geriatric KT strategy. Many of the necessary building blocks for effective KT are now in development, including the expansion of infrastructure, education and training of healthcare providers and informal caregivers, and the development of geriatric research institutes. The NPHCE offers an unprecedented opportunity in India to improve elder care and prepare for the future needs of an aging population. This paper argues that we can enhance the probability of success and impact of the NPHCE through the development of a geriatric KT action plan that incorporates and applies theories, frameworks, and evidence for activating the knowledge-to-action cycle.

## Competing interests

The authors declare that they have no competing interests.

## Authors' contributions

JE constructed the first draft of the manuscript with subsequent inputs and revisions from OB and PK. All authors read and approved the final manuscript.
